# Criminal Abortion

**Published:** 1860-10

**Authors:** 


### Criminal Abortion.

We find in the September number of the "American Medical Monthly and New York Review," the "Report of a Trial for Criminal Abortion. By C. P. Frost, A. M., M. D., of St. Johnsbury, Vt." It seems that the service of an Irishman, calling himself Dr. W. H. M. Howard, was engaged, for the sum of $100, to destroy the fruit of an illicit connection between a young farmer and his servant girl. The attempt was successful; and the result was, that the silly girl also died from the effects of the means used to destroy her unborn child. The self-styled Doctor is now undergoing but the mere name of punishment, for so monstrous a crime, by confinement in the State prison for two years.

It appears, also, from the Report of Dr. Frost, alluded to, that there was in the Bill of Indictment also a charge of manslaughter, of which, however, he was acquitted by the jury. An indictment found at the Court in which he was convicted, in another case, for the same offence, awaits the expiration of his sentence. If even the meagre justice rendered him already, should be meted him in the other case, the wicked ones of Vermont, desiring to commit *murder* upon the innocent fœtus in utero, will be compelled, for four years at least, to employ some other agent.

This crime, we fear, is not looked upon with the horror its importance deserves, in many parts of the country, even by those occupying high positions in society. When females are sustained by public sentiment and retained as members of respectable society, who indulge the desire to destroy the embryo or fœtus in utero, for no other reason than the trouble of raising, we say the moral status of the country is at a low ebb, and we fear this is the case in more instances than is generally known.

